# Plastic Surgery

**Published:** 1868-05

**Authors:** 


### BIBLIOGRAPHICAL NOTICES.

---

*Plastic Surgery.*—By David Prince, M.D. Publishers, Lindsay and Blakiston, Philadelphia. This monograph, containing a new classification and a brief exposition of Plastic Surgery, is well written and will prove a valuable addition to the library of the dental, as well as to that of the medical practitioner. Its contents consists of a Definition of Plastic Surgery, with the General Hygienic Conditions favorable to success, Varieties, Cicatrix, Rhinoplasty, Otoplasty, Blepharoplasty, Operations Involving the Mouth, Extroversion of the Bladder, and Autoplasty of the Penis. The chapter on "Operations Involving the Mouth," including Uranoplasty, or Palatoplasty, Hare-lip and Cheiloplasty, is a very interesting one, and contains much that is useful in dental practice. Referring to the objections, of many operators to administer general anæsthetic agents upon occasions of operations about the mouth, through fear of the entrance of blood into the air-passages, an operation, by the way, of daily occurrence in dental practice, the author speaks as follows :

"The immunity from any evil consequences from the entrance of blood into the larynx, in the following cases, and the writer's uniform practice of giving anæsthetics, in operations about the mouth, encourage the belief, that the entrance of blood into the larynx, under ether, is altogether chimerical." "Blood is readily removed by coughing, as the patient rouses, and if a clot should possibly close the glottis, the "ready method" would immediately displace it, either upward, or downward, as the patient rolls over, filling and emptying the lungs." "The rolling of the patient over, in the horizontal position, being the method which it is imperative to employ, upon the least alarm, while the patient is in a state of anæsthesia, renders it impossible that a blood clot should resist the force of the atmosphere, as it enters and leaves the chest with the expansion and contraction of the ribs." "The local irritation of blood can be no more than that of so much mucus, as is shown by cases of hæmoptysis." "The chief objection, therefore, to the employment of anæsthetics, while operating about the mouth, is the necessity for occasionally suspending the proceedings, to administer the anæsthetic."

The work is well gotten up and presents the neat appearance common to all the publications of Lindsay and Blakiston.

